# ‘The weight of the world on your shoulders’: Nurses’ perceptions – good practices and challenges in asylum seekers’ initial health assessment

**DOI:** 10.1177/17449871251386244

**Published:** 2025-12-03

**Authors:** Katri-Leena Mustonen, Jaana Tilli, Awa Ahmed Haji Omar, Tomi Mäki-Opas, Anu Castaneda, Jussi Kauhanen, Natalia Skogberg

**Affiliations:** Senior Planning Officer, Department of Healthcare and Social Welfare, Finnish Institute for Health and Welfare & Institute of Public Health and Clinical Nutrition, Faculty of Health Sciences, University of Eastern Finland, Finland; Senior Lecturer, Diaconia University of Applied Sciences & Faculty of Health Sciences, University of Eastern Finland, Finland; Project Coordinator, Department of Healthcare and Social Welfare, Finnish Institute for Health and Welfare, Finland; Professor, Faculty of Social Sciences, University of Eastern Finland & North Savo Wellbeing Services County, Wellbeing Services Research Centre, Finland; Research Professor, Department of Healthcare and Social Welfare, Finnish Institute for Health and Welfare, Finland; Professor, Institute of Public Health and Clinical Nutrition, Faculty of Health Sciences, University of Eastern Finland, Finland; Chief Researcher, Department of Healthcare and Social Welfare, Finnish Institute for Health and Welfare, Finland

**Keywords:** asylum seekers, needs assessment, nurses, professional practice, quality of care, refugees, trust

## Abstract

**Background:**

Annual asylum applications in Europe have exceeded one million, highlighting asylum seekers’ unique health needs and the increasing importance of refugee health nursing practices. In Finland, all asylum seekers are offered a free and voluntary initial health assessment conducted by nurses at reception centres.

**Aim:**

To identify good practices and challenges in asylum seekers’ initial health assessment from nurses’ perspectives.

**Methods:**

Participants were selected via purposive sampling. Between January and February 2019, we conducted 14 semi-structured interviews with reception centre nurses performing the initial health assessment. Data underwent thematic analysis.

**Results:**

Three themes were identified: (1) building a relationship of trust between nurse and client, (2) interprofessional collaboration, and (3) continuity of care. Each theme included a client-centred approach. The findings reflect client, professional, and system levels, which can be located on a timeline from first contact to follow-up care.

**Conclusion:**

Building trust is a foundational step, achieved through a client-centred approach, tailored interactions, and the provision of information. Clarifying the nurses’ role benefits both interprofessional collaboration and professionals’ well-being. Due to fragmented health records, precise documentation is essential for ensuring continuity of care. These findings can inform service development and professional training.

## Introduction

Annual asylum applications in Europe have exceeded one million, emphasising the growing need for refugee health nursing (Desmyth et al., 2021; European Union Agency for Asylum, 2025). It is a human rights-based specialty that addresses the unique health needs of asylum seekers and refugees. Asylum seekers are persons who seek international protection and whose application for refugee status is being processed (United Nations High Commissioner for Refugees [UNHCR], 2006). Rights to health and seeking international protection are safeguarded by the Universal Declaration of Human Rights (United Nations 1948/2020). The Recast Reception Conditions Directive establishes asylum seekers’ right to Europe-wide healthcare services, but member states determine the scope of its implementation (European Commission, 2023). For example, initial health assessments (IHAs) are conducted to varying extents (Jonzon et al., 2018). IHAs and resulting interventions aim to address the complex needs of individuals, benefiting both their health and integration as well as contributing to public health and economic outcomes for society (Frederiksen et al., 2013). While increasing epidemiological data on asylum seekers’ health and their lived experiences are gradually helping to inform the development of optimal health services (Obtinario and Thorkildsen, 2020; Skogberg et al., 2019), yet, according to Gold et al.’s (2025) systematic review, research on models of care for refugees and the role of specialised nurses in implementing targeted measures remains limited. Nurses, among other professionals on the frontline, must overcome challenges at individual, professional and health-system levels to be able to provide the same quality of care as for the general population (Robertshaw et al., 2017).

Individual-level considerations involve asylum seekers’ perspectives, including factors affecting migrants’ health, cultural health beliefs, and health literacy. Their health is affected in three phases: pre-migration, during the journey, and in the receiving country. In addition to individual and environmental factors, several elements influence migrants’ health (Spallek et al., 2011; World Health Organisation [WHO], 2021). Prior to departure, the state of the healthcare system and the reasons for fleeing the country of origin can significantly impact health. During the journey, individuals may be deprived of necessities and healthcare and may face exploitation and potentially traumatic experiences. In the country of destination, challenges related to the migration process – along with experiences of inequality and discrimination – can further affect health outcomes. Among all migrant groups, refugees are particularly vulnerable (Grove & Zwi, 2006; Gostin & Roberts 2015, Hossin, 2020). According to the Asylum Seekers’ Health and Well-being Survey, the most common long-standing somatic diseases were cardiovascular, musculoskeletal, and respiratory (Skogberg et al., 2019). Sexual and reproductive health problems were frequently reported by women. Almost 50% adult participants suffered from permanent injuries, and oral health issues were frequent. In addition, 83% had experienced at least one potentially traumatic event and 40% adult participants reported symptoms of severe depression and anxiety (Skogberg et al., 2019). Cultural beliefs related to health influence individuals’ perception of illness, their engagement with healthcare services, and their commitment to treatment (Berkman et al., 2011). Health literacy refers to the ability to understand and apply health-related information (Wångdahl et al., 2014). Among asylum seekers, limited health literacy may hinder the effectiveness of health assessments (Wångdahl et al., 2015). According to Koehn’s (2005) study in Finnish reception centres shared understanding of key elements of health increases asylum seekers’ satisfaction with healthcare appointments.

To effectively and equally address asylum seekers’ needs, competent professionals are essential. The WHO (2021) has established global competency standards for health workers in refugee and migrant health. However, these standards need to be aligned with practical delivery guidelines. Key considerations for newly arrived refugees, according to Van Loenen et al.’s (2018) study investigating primary care, comprise language barriers and the need for a culturally competent healthcare workforce. Cultural competence refers to a specific set of skills, knowledge and an awareness of the impact of one’s own cultural context (Desmyth et al., 2021; Sharifi et al., 2019). In addition, for nurses working with asylum seekers, specific competences include a knowledge base on the health effects of migration, being able to use a trauma-informed approach, and the ability to communicate how the healthcare system functions in the receiving country (Suurmond et al., 2010). The majority of asylum seekers have experienced potentially traumatic events, which may affect their interactions with health services (Skogberg et al., 2019; WHO, 2021). Therefore, it is recommended to incorporate knowledge and understanding of how trauma impacts individuals – that is, a trauma-informed care approach – without delving deeply into the specific events. The nurses support for this group extends to social and logistical aspects alongside personal health needs.

In Finland, adult asylum seekers are entitled to urgent and essential care, the latter assessed by a licensed healthcare professional (Act on the Reception of Persons Applying for International Protection and on the Identification of and Assistance to Victims of Trafficking in Human Beings 746/2011, later referred as the Reception Act). Furthermore, asylum seekers have the right to maternal and child health clinic services and school healthcare (Finnish Institute for Health and Welfare, 2023). Asylum seeker children’s entitlement to care is the same as that of children with permanent residence in Finland. In addition, the Reception Act (746/2011 § 6) requires identifying persons in vulnerable situations and resulting special needs. The Finnish Immigration Service is responsible for organising and overseeing such reception services (Finnish Institute for Health and Welfare, 2023). In the current system, during the asylum process, healthcare services are provided by the reception centre, which coordinates these services in collaboration with the public sector, through contracts with private service providers, or both.

The IHA includes a health information session, interview, health examination, screening tests, and vaccinations. It is conducted by reception centre nurses, and based on the assessment, further care is provided. These nurses work as part of an interprofessional team that includes social workers and social advisors. In addition to assessing health service needs, these nurses are responsible for coordinating each asylum seeker’s healthcare plan in collaboration with maternity services, child health clinics, school care, hospitals and the private sector (Finnish Institute for Health and Welfare, 2023).

Despite the unique healthcare needs of this group high-quality care is ethically vital. The Organisation for Economic Co-operation and Development [OECD] (2022) defines quality care as patient-centred, safe, and effective. The relationship between Input, Output, Outcome, and Effectiveness can be illustrated by the Chain of Effectiveness (Liket, 2017). Good practices (Output) are actions designed to produce desired outcomes and achieve specific aims. In addition to good clinical practices, we need to adopt practices that enhance patients’ perceived quality of care, in this context migrant health brings unique aspects for consideration (Hanefeld et al., 2017). Perceived quality is a multidimensional concept, encompassing factors beyond traditional indicators such as health service coverage, service utilisation, and outcomes. It involves elements that influence whether patients seek treatment, communicate their service needs, and continue with treatment. In essence, it provides a comprehensive appraisal of the treatment process. Patients’ experience of care and perceived quality is significantly shaped by their interactions with healthcare providers.

## Methodology

This study aimed to identify good practices and challenges in newly arrived asylum seekers’ IHA from nurses’ perspectives. We aimed to contribute to understanding nurses’ work in the reception centre context. To achieve these aims, we employed an interpretive paradigm enabling the recognition of nurses’ perspectives and acknowledging that client/patient care is context-dependent (Weaver and Olson, 2006).

### Participants

Inclusion criteria comprised working in reception centres and performing IHAs for asylum seekers in Finland. Using purposive sampling (Campbell et al., 2020), we approached such nurses for their specialised knowledge. All nurses fulfilling these criteria were invited to participate in the study, which was promoted in their meetings and via e-mail. At the time of participant recruitment, 97 nurses were working in Finnish reception centres; of these, 14 volunteered to participate. However, the number of nurses actively conducting IHAs is limited to those working in transit reception centres. Due to the small number of reception centre nurses, to protect anonymity, we did not gather personal demographic details. According to the interviewer’s observations, the majority of these 14 had public health nurse degrees and had worked in reception centres more than a year but less than 5 years.

### Data collection

To explore nurses’ perspectives, we formulated an interview guide (Appendix 1) adapted and modified from the recommended practices identified in previous research and development projects concerning migrants. The guide included four themes: (1) the primary health examination as a phenomenon, (2) good practices in the primary health examination, (3) challenges in the primary health examination and (4) development needs and proposals for the new primary health examination protocol. The interview guide was not piloted, and no modifications were made during the course of the study. During January and February 2019, the first author, who holds a Master’s degree in Health Care, conducted semi-structured interviews. Of the 14 interviews, all of which were recorded, 13 were conducted face-to-face, mainly at participants’ workplaces; the remaining 1 was by telephone. Each nurse was individually interviewed in Finnish by one interviewer, who also simultaneously prepared field notes. Interview length ranged from 36 to 98 minutes.

### Data analysis

These interviews were transcribed verbatim. However, transcripts were not provided to the participants due to the rapid pace of the project. Preliminary findings were utilised in a workshop where all target groups – asylum seekers, nurses, and authorities – had the opportunity to comment on and discuss collaboratively, contributing to IHA further development. We used Braun and Clarke’s (2006) thematic analysis (TA) framework due to its accessibility for early career researchers and theoretical adaptability. TA is a systematic approach to data analysis, resulting in themes that comprehensively (re)present nurses’ perceptions.

TA consists of six phases. First familiarisation with the data; the data were read through several times and notes prepared. Next code generation; open coding was employed, and codes related to good practices and challenges were separately identified. Third, the generation of initial themes; the data were clustered from small items to prominent entities, and related entities were grouped into sub-themes. Fourth, reviewing of initial themes; an initial chart of the findings was created, and the logic/flow was iteratively reviewed by the research team. Fifth, defining and naming themes by the team and consensus reached. The last step is report production. This is presented below according to themes and subthemes identified.

During the analysis phase, data interpretation was regularly cross-referenced with the interview transcripts. However, the interviews and analysis were conducted in Finnish; thus, accuracy and authenticity may have been slightly compromised in the process of translation into English for the present paper.

### Ethical considerations

This study was performed in line with the Declaration of Helsinki principles. Ethical approval was obtained from the regional ethics committee of the Helsinki and Uusimaa hospital district (3306/2017). All participants signed a written consent form and received written and verbal information about the study; they assented to their interviews being recorded. Participants were informed of their right to withdraw from the study.

## Findings

**Figure 1.**
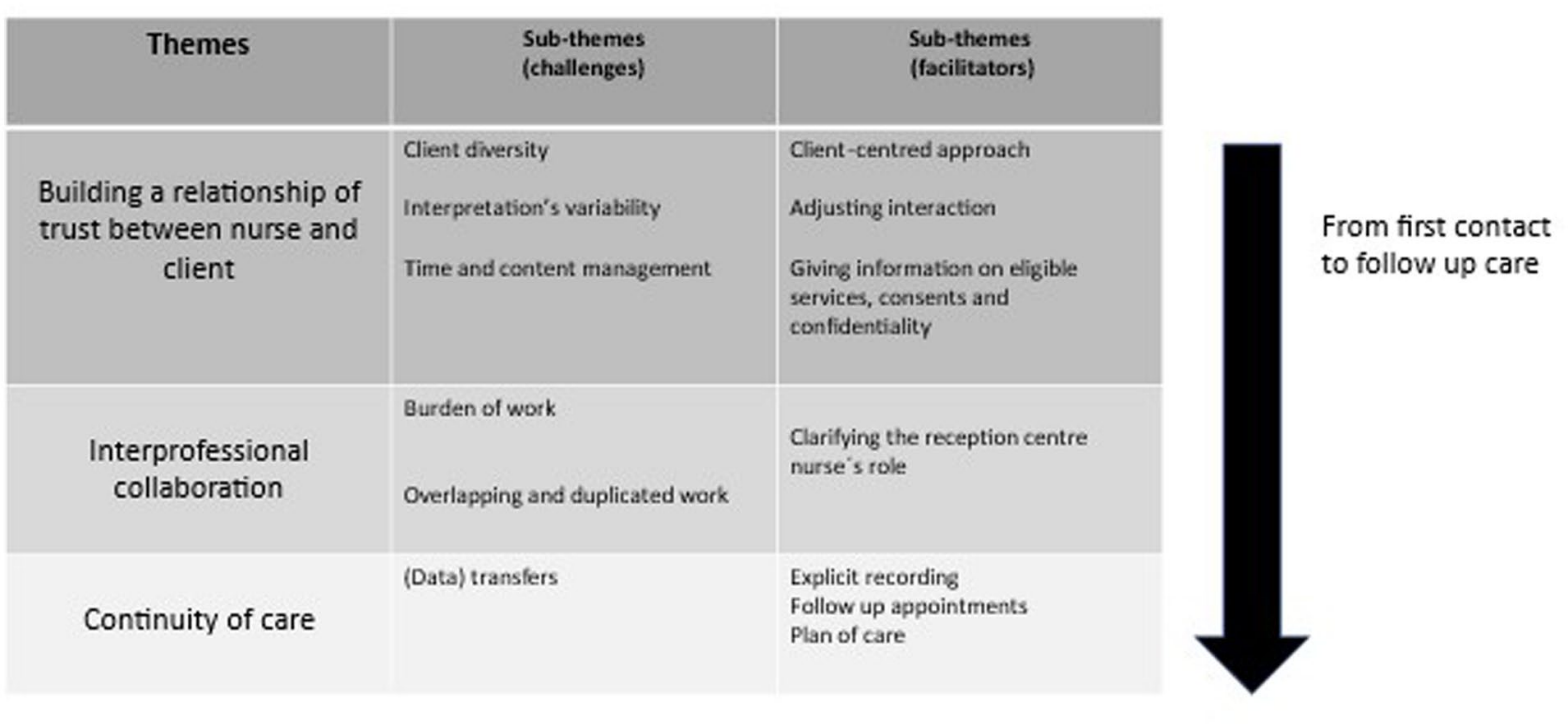
Thematic summary of the findings.

The findings of the study are organised under three themes: building a relationship of trust between nurse and client, interprofessional collaboration, and continuity of care. Each theme has sub-themes that describe challenges and facilitators (Figure 1).

### Theme 1: Building a relationship of trust between nurse and client

Interviewee 1 at reception centre 7 summed up the first theme: ‘*the most important aim of the initial health assessment is to gain trust*’. Challenges related to this theme were client diversity, interpretation variability, and managing IHA content and timeline. Adopting a client-centred approach, adjusting interaction and providing information about services, consent and confidentiality facilitated creating a relationship of trust.

### Challenges in theme 1: Building a relationship of trust

#### Client diversity

Nurses described client diversity, including differences in education and vulnerability levels, as both a challenge and an asset: ‘With some people, things go very smoothly, but of course, there are times when challenges arise – sometimes stemming from the client’s cognitive abilities, making it difficult to fully engage, or at times, from an inability to build a proper connection.’ (Interview 2, reception centre 5)

Although some clients did not participate in the IHA, others participated but were reluctant to share their thoughts. Additionally, some clients faced difficulties in understanding key concepts, such as data storage and consent. Nurses often struggled with how to present information in a manner that ensured mutual nurse–client understanding. Clients’ varied backgrounds also influenced the types of questions that nurses asked. Moreover, some interviewees highlighted the challenge of managing clients who requested services for which they were ineligible.

#### Interpreter variability

Nurses reported challenges in building a relationship with clients when working with an interpreter, particularly when this is not an official interpreter as the quality of interpretation and the lack of professionalism among some interpreters were perceived as problematic: ‘*Sometimes there are situations in which the client starts crying and then the interpreter starts crying too. In that situation, as a nurse I have to support both of them . . . it would always be good to have the education, to have professional interpreters. Then the person doesn’t start telling their own stuff. Some interpreters cannot handle the silence at all. If you ask the client how they are feeling and the person is just quiet, then you should give them time and space and not watch the clock thinking when does this end . . . because of getting anxious.*’ (Interview 1, reception centre 4)

The use of specialised terminology and jargon further complicated the interpreting process. Some nurses perceived that clients found it easier to discuss sensitive matters with an interpreter of the same gender. They also noted that when the interpreter and the client failed to understand each other during an encounter, rescheduling another appointment proved difficult.

#### Time and content management

The nurses emphasised that building a trusting relationship requires time, active listening, and in-depth interviewing, all of which can create challenges in managing both time and content effectively, especially in a clinic environment with scheduled time-limited appointments. ‘*To pull yourself together and, all of a sudden, in an hour, go through another person’s life and put it sensibly on record — that is a challenge.*’ (*Interview 3, reception centre 5*)

The lack of a standardised template meant that each nurse had to prioritise independently. Some participants identified time constraints – such as the 2-week time frame as specified in the guidelines and the 30–60-minute allocation per client – as significant challenges, particularly when dealing with large families who prefer a single appointment for the entire family. Variation in assessment duration, influenced by the client’s needs or the nurse’s focus, was also viewed as an equity issue. In some cases, nurses chose to limit assessment content, due to the client’s situation, such as a lack of emotional capacity. A common challenge among interviewees was the need to tackle numerous important topics with client ability to cope. For some nurses, who only carried out IHAs occasionally, lack of proficiency in the process was difficult, further exacerbating challenges related to prioritisation and time.

### Facilitators in theme 1: Building a relationship of trust

#### Client-centred approach

Nurses emphasised the voluntary nature of participation and the importance of client-led dialogue, recognising that a client-centred approach is essential for building trust. Rather than pressing for disclosure, they respected each client’s autonomy in deciding what information to share. As a result, while interviews alone may not always reveal infectious diseases, the establishment of trust through respectful, client-driven interactions was seen as a more effective foundation for long-term engagement and disclosure: ‘*These appointments follow a client-first approach. If there are serious health issues or concerns, they are addressed first. Otherwise, if everything is fine, the initial health assessment (IHA) doesn’t take long.*’ (Interview 1, reception centre 2)

Therefore, even when the IHA had a structured approach, nurses acknowledged the need to tailor this structure to individual clients. They strive to provide clients with the opportunity, space, and time to discuss even difficult topics, without applying pressure, and instead encourage clients to return if necessary. These nurses also stressed that, although subsequent care is based on the IHA, clients are ultimately responsible for managing their own health concerns. However, they recognised that clients may initially struggle to articulate their needs or understand their rights and building a sense of trust through a client-led approach was essential to optimum care.

#### Adjusting interaction

The nurses emphasised the importance of adapting to each situation by considering clients’ level of understanding and adjusting nurses’ questions, language and interactions accordingly: ‘*Of course, one thinks about our own performance. These kinds of encounters need to be evaluated, how they could be better brought to the client’s level.*’ (Interview 2, reception centre 5)

Nurses emphasised that they fine-tune their communication with small gestures and carefully assess whether to proceed with sensitive questions or ask follow-up questions. They respected their colleagues’ individual personalities and were attentive to clients’ reactions. The nurses aimed to create a pleasant, positive and warm initial encounter, noting that humour can be mindfully used. The ability to create a relaxed atmosphere is often achieved through experience and an understanding that rushing is unnecessary, especially when dealing with clients who have experienced trauma. Listening attentively was highlighted as crucial, even when they may be unable to directly influence the client’s situation.

#### Giving information on eligible services, consent, and confidentiality

Nurses underlined the importance of providing comprehensive information about eligible services, particularly when clients are unfamiliar with the local healthcare system, and of obtaining informed consent for recording and data transfer. It was vital to establish these understandings at the outset of the IHA. They raised how confidentiality and the legal obligation to secure consent for data transfer can foster trust in some clients but may also provoke suspicion in others. There were challenges in helping clients understand their entitlements and legal limitations on services, with some expressing the need to avoid creating false expectations: ‘*One has to keep in mind to which services they are entitled. Also, the quality of the service has to be stable for everyone, so no one receives more than others.*’ (Interview 2, reception centre 1)

The importance of justifying questions posed during the assessment and reassuring clients that their consent information would remain unshared between professional groups or authorities. was stressed by participants However, they acknowledged that, in some cases, allowing such transfers of information might be in clients’ best interests. Additionally, these nurses stressed the importance of informing clients on how to proceed after the IHA and how to contact them if further assistance is needed.

### Theme 2: Interprofessional collaboration

Challenges related to this theme are work burden as well as overlapping and duplicated work. To tackle these challenges, clarifying reception centre nurses’ role relative to other professionals was viewed as an Interviewee 1, reception centre 2, describes interprofessional collaboration benefits: ‘*Maybe one tries to seek some kind of professional support to their own decision-making from another point of view’.*

### Challenges in theme 2: interprofessional collaboration

#### Burden of work

The nurses emphasised that their work in reception centres is particularly demanding, especially in transit units with rapid client turnover. They must manage the care of hundreds of clients while maintaining sensitivity to spend time and focus on individual needs. They described their workload as urgent, unpredictable, and plagued by a persistent sense of inadequacy. Balancing detailed administrative tasks, particularly with constantly changing client populations, alongside the profound life tragedies and global issues that clients face, was described well in Interview 1, reception centre 2: ‘*You can’t do everything, carry the weight of the world on your shoulders’.*

Some nurses felt pressured to remember every detail that might influence individuals’ asylum process, yet they often found themselves with limited ability to affect outcomes. To address feelings of uncertainty and haste inherent in their work, nurses employed two key practices: scheduling various appointment times in advance through the calendar and effectively informing clients about these appointments and other available services.

#### Overlapping and duplicated work

Due to incomplete records and poor communication between reception centre nurses, tasks often had to be repeated, particularly when clients transitioned quickly from transit centres to long-term facilities: ‘*I can’t know everything. Then I have to ponder, then I have to call the polyclinic. I’m calling about this. In a way, it increases the amount of work quite a lot, you must do something because of incomplete records. . .Well, and in the best or worst case, there is always a third nurse who calls and asks about the same thing.*’ (Interview 4, reception centre 4)

Several interviewees noted that the same tasks were often repeated by different professionals across health services, that is, doctors’ appointments, school health and hospital care. Additionally, uncertainty about the content of social workers’ appointments led nurses to explain the service system beyond just health services. Some participants checked children’s vaccinations and reported them to child health clinics, whereas others felt that this work was beyond their remit.

### A facilitator in theme 2: Interprofessional collaboration

#### Clarifying the reception centre nurse’s role

The IHA was described as central to their work. The qualities of effective reception centre nurses were discussed. Essential traits included mastery of clinical skills, relevant experience with diverse populations, and a firm and resilient temperament – someone who is able to set clear boundaries while remaining calm and composed under pressure. They also emphasised that skills should be practically applied and tailored to clients. In some reception centres, background education and experience influenced job duties. Nurses’ core responsibilities were identified as identifying infection symptoms, assessing the need for screening tests, and checking for Bacillus Calmette-Guérin scars. Understanding clients’ backgrounds and health-related issues was crucial, as was managing clients’ subsequent care, including guidance, support, and coordination of follow-up care and examinations. IHA nurse roles also involved preparation for other health professional’s appointments. Passing on relevant information was essential. Regarding the social worker and asylum process, nurses were expected to map the travel route from a health perspective and clarify their care plan. In some centres, healthcare secretaries handled basic record-keeping and counsellors managed appointment bookings, allowing nurses to focus on critical nursing roles. Participants felt that only qualified professionals should assess appointment urgency. Some participants noted that reviewing hospital instructions was outside the nurse’s role since clients reside in reception centres. In other words, clients should be approached at the time of hospital discharge in a manner that supports their ability to manage independently. Ultimately, client variety rendered the work engaging; compared to other public health nurses, reception centre nurses expressed a strong sense of belonging to their professional community: ‘*The job of a public health nurse at a reception centre is kind of about everything, so you can’t really say that a public health nurse is the professional of prevention, but we have so much stuff like (you are) a nurse and sometimes it feels that you are almost a psychiatrist [laughs] and a public health nurse and a little bit of a midwife [laughs].*’ (Interview 1, reception centre 5)

### Theme 3: Continuity of care

Nurses saw poor information transfer as a risk to care continuity. The facilitators in this theme were explicit recording, ensuring follow-up appointments as indicated, and a clear care plan to ensure that: ‘*The next place would already know to organize the necessary help, if there is something needed.*’ (Interview 1, reception centre 4)

### A challenge in theme 3: Continuity of care

#### (Data) transfers

The temporary nature of transit reception centres, through which clients can move quickly, poses unique challenges. Nurses highlighted the importance of transferring accurate and complete information to subsequent services. A significant issue is the lack of standardised protocols across reception centres, leading to uncertainty about assessment and care for individual clients. Additionally, incompatibility across computer systems increases the risk of failure in communicating information, particularly when information is not recorded or when record quality is poor: ‘*The challenge is precisely that computer programmes are not compatible, and it leaves gaps in the data transfer.*’ (Interview 1, reception centre 2)

Nurses noted that, in the current client/patient record system, documenting appointment content can be challenging and recording laboratory results and making referrals can be time-consuming. Incomplete records were also flagged as a potential client/patient safety concern.

### Facilitators in theme 3: Continuity of care

#### Explicit recording

The importance of comprehensive documentation was raised. The nurses suggested that records should clearly indicate whether the IHA was completed as well as questions asked, answers provided, and screening conducted: ‘*Over time, I have developed my own formula about what to ask and what not to ask. Sometimes I have looked into others’ notes just to see how this person has written about the same subject. It’s just unbelievable how people can write in such different ways and what they emphasise – is it physical, psychological or social ability to function . . . What is then emphasised or is it trivial and are all blood pressures and all weights in there.*’ (Interview 3, reception centre 4)

Ideally, standardised recording would allow nurses at subsequent reception centres to seamlessly continue care based on previous records. Such continuation is particularly crucial when working with vulnerable clients. The nurses believed that good initial documentation could eliminate the need for unnecessary appointments to determine follow-up (actions), saving time and resources. They also highlighted the importance of recording sensitive information, such as female genital mutilation, which may have an impact on the asylum process. Additionally, they stressed the need for equal treatment, ensuring that all clients have the same baseline records. It was stressed that clear and detailed documentation benefits collaborative interprofessional working.

#### Follow-up appointments

Follow-up appointments served several purposes: in case a need for discussion arises as the IHA appointment has been used to handle acute issues; monitoring is necessary for clients starting new medication or, for example, when blood pressure needs checking, etc.; physical measurements/follow-up was needed to observe those in vulnerable situations; to facilitate building a relationship of trust over time: ‘*Perhaps there is not much more you can hope from that first contact than that a person was left with the feeling that hey, this day is over and probably not everything was talked about between earth and heaven, but the feeling was left that I can be in contact later.*’ (Interview 1, reception centre 3)

#### Plan of care

These interviewees stated that care plans are central in nursing and the IHA. At the end of an IHA, clear care plans identifying requirements and the professional responsible for follow-up care is important for a clear picture that can be followed by any healthcare professional – ensuring continuity of care: ‘*You can tell (that the aim is met) by the fact that there is a smart plan for a client, it clearly states what happens next. Or it is that there is no need for anything acute and the client will contact you, if necessary, that’s fine . . . That plan is the final summary of what needs to be done based on the appointment.*’ (Interview 2, reception centre 2)

A plan of care was also important to indicate that health assessment objectives have been met. For some of the nurses, a missing action plan rendered the recording the whole IHA as unproductive.

## Discussion

The aim of this study was to examine good practices and challenges in asylum seekers’ IHAs, from the perspective of reception centre nurses, and to contribute to knowledge on refugee health nursing practices (Desmyth et al., 2021). Three key themes were identified: (1) building a relationship of trust between nurse and client, (2) interprofessional collaboration and (3) continuity of care, along with the associated barriers and facilitators for each. Firstly, a client/patient–nurse relationship must be established. Interprofessional collaboration requires cooperation among professionals, and continuity of care involves the transfer of information between reception centres and various healthcare facilities. These themes operate on three levels: from client to professional, between professionals and from professionals to organisations. They also span the timeline from initial contact to follow-up care. Good practices help address challenges related to asylum seekers’ complex health needs, intercultural communication, and structural barriers, ultimately contributing to the identification of service needs and care quality.

Asylum seekers’ lack of trust often stems from previous life experiences, presenting a significant barrier to accessing healthcare. Establishing trust is crucial for accurately assessing service needs and integrating clients into the national healthcare system. Nurses emphasised the importance of adjusted interactions, client/patient-centred approaches, and the provision of clear information. This finding aligns with prior research, which highlight how lack of information, communication challenges, and time constraints are central factors affecting healthcare access among refugees (van Loenen et al., 2018). Moreover, increasing trust enhances perceived quality of care (Legido-Quigley et al., 2015). When evaluating different models of care, as recommended by a recent systematic review (Gold et al., 2025), it is important to consider the types of systems that enable trust-building between healthcare providers and patients. This involves comparing models not only in terms of efficiency and outcomes but also in their capacity to foster meaningful, trust-based relationships.

Furthermore, according to Gold et al. (2025), specific knowledge on nurses’ role in refugee healthcare models, especially in primary care settings, is limited. Our study shows that nurses’ workload challenges and the potential for redundant tasks could be tackled by clarifying their roles relative to those of other professionals. Effective interprofessional collaboration was identified as enhancing care comprehensiveness, supporting decision-making, and ensuring teamwork that benefits clients, particularly in complex cases such as victims of human trafficking. Such collaboration ideally leads to improved precision in identifying client/patient needs and enables nurses to focus on their specialised skills, contributing to high-quality and client-centred care (Dubois and Singh, 2009; Lavander et al., 2017; WHO, 2010).

IHAs serve as a critical first point of contact for asylum seekers within the Finnish healthcare system, rendering continuity of care essential. Gold et al. (2025) noted that understanding of the role of nurses in ensuring such continuity was limited. The theme, care continuity, closely aligns with the previous two: trust in individual encounters and smooth interprofessional collaboration form the foundation for ensuring care continuity. Consistent information transfer and robust documentation practices are crucial for ensuring seamless care transitions and continuity, particularly given asylum seekers’ mobility between reception centres and the fragmentation of their medical data due to their exclusion from the Finnish digital health information system. Lack of continuity significantly impedes healthcare access for refugees across Europe (van Loenen et al., 2018). Improving continuity is likely to enhance perceived quality of care (Hanefeld et al., 2017).

### Strengths and limitations of the study

This study had 14 (volunteer) respondents, who represented the majority of the nurses actively performing IHAs in different locations. To mitigate the risk of absent voices, we employed purposive sampling. These nurses seemed genuinely interested in participating in a study that was aimed at developing IHAs. However, some of them may have felt pressure to act as workplace representatives. The interviewer worked in the development project and had a background in reception centre nursing; this could have had both a positive and negative impact. In-depth knowledge of the project and understanding of reception centre nurses’ work may have facilitated trust building and interviews, while increasing credibility. On the other hand, participants’ responses may have been influenced: the interviewer was not an external researcher; therefore, participants may not have expressed everything in detail. Nevertheless, that one person, who was able to gain a truly insider perspective, conducted all the interviews and led the analysis is a strength to the study as she had experience and training, having previous experience in conducting qualitative study. The transcription was prepared by a professional company. Although this supported the efficiency of the process, it may have initially compromised immersion in the data. The findings section (including extracts) was proofread by a native English speaker to mitigate the risk that a translation from Finnish to English may lead to the loss of subtle nuances, cultural context, and specific meanings.

### Recommendations

This study contributes to understanding refugee health nursing practices, by focusing on three areas: First comes increasing trust through client-centred care, tailored interactions, and clear information provision. Next is applying interprofessional collaboration to allow nurses to concentrate on their specialised roles. The third is improving documentation systems and protocols, to ensure continuity of care and facilitate follow-up appointments. These practices should inform the development of services and training programmes for professionals working with asylum seekers. Despite different service system types, these findings can help in training nurses within parallel systems and in private or public healthcare systems. The reality is that increasing numbers of nurses will encounter clients/patients with refugee backgrounds.

Carrying the weight of the world on one’s shoulders can be emotionally and mentally exhausting. Addressing this requires targeted training, supportive materials, and reflective supervision. The aforementioned good practices, along with a standardised protocol and accompanying handbook, may help alleviate the burden faced by nurses in reception centres. Notable examples include the Psychoeducation and Mental Health Promotion for Newly Arrived Refugees (TUULI) materials and training programmes such as PALOMA, which provide professionals with tools to engage effectively with individuals from refugee backgrounds and support their well-being (Finnish Institute for Health and Welfare, 2024a, 2024b).

The chain of effectiveness framework highlights the need for further research. Firstly, we need to clarify IHA objectives from the perspectives of asylum seekers, reception centre nurses, and health authorities. Secondly, to emphasise the unique expertise of reception centre nurses, further studies should investigate competencies aligned with IHA aims. Given the potential for absent voices in this study, a future electronic survey could aim to capture perspectives that may have been underrepresented or omitted. Furthermore, outcomes should be analysed at client/patient, professional, and systemic levels. Finally, IHA effectiveness should be evaluated by examining aim achievement.

## Conclusion

The IHA has multi-level effects on individuals, professionals, and society. To ensure high-quality care, the adoption of evidence-informed refugee health nursing practice is essential. The present study contributes to a deeper understanding of nurses’ roles in this context, particularly given the limited prior research and the unique needs of this client group. By seeking expert consensus, we aimed to identify practices that are both effective and applicable, allowing limited resources to be directed towards interventions with the greatest impact and helping ensure smoother care pathways for clients.

**Key points for policy, practice and/or research**

- Health policies should mandate the evaluation of models of care based on their ability to foster trust-based relationships, particularly with refugee and asylum-seeking populations.
- Evidence-based refugee health nursing practices are needed in a world with increasing numbers of refugees and asylum seekers.
- The outcomes and effectiveness of the IHA model should be analysed at the client/patient, professional, and systemic levels.

## Appendix 1

### Interview Guide: Reception centre healthcare professionals’ thematic interview

#### Theme 1: Description of the phenomenon

– What do you think are the main aims of the initial health assessment?
– Do you have an initial health assessment template in use? (If yes: What kind of? Please provide.)
– Have you been instructed in performing an initial health assessment? (If yes: What kind of? From whom?)
– Do you have written instructions for performing an initial health assessment? (If yes: Produced by whom? Please provide.)

#### Theme 2: Good practices

– What are the key issues you always address during the initial health assessment?
– What kind of things should not be included in the initial health assessment?
– How do you identify those in vulnerable position?
– What kind of things facilitate building a relationship of trust with the client?
– How do you know that the initial health assessment has met the aims you mentioned earlier?

#### Theme 3: Challenges

– What do you think are the biggest challenges in conducting an initial health assessment?
– How do you intend to address these challenges/how do you think they could be addressed?

#### Theme 4: Development needs and suggestions

– What themes do you think the TERTTU initial health assessment model should include?
– How would you improve the use of existing resources in the implementation of the initial health assessment?
– What other ideas would you have for developing the initial health assessments?
– How has the TERTTU project influenced your work? Have you received enough information about it/what kind of information would you like to receive about this project?
– What kind of in-service training would you like to receive in order to be able to perform initial health assessments better than before?
